# High HIV-1 diversity in immigrants resident in Italy (2008–2017)

**DOI:** 10.1038/s41598-020-59084-2

**Published:** 2020-02-24

**Authors:** Maria Teresa Maggiorella, Nunzia Sanarico, Gaetano Brindicci, Laura Monno, Carmen Rita Santoro, Nicola Coppola, Nunzia Cuomo, Annalisa Azzurri, Francesco Cesario, Filippo Luciani, Issa El-Hamad, Gabriella D’Ettorre, Ombretta Turriziani, Laura Mazzuti, Alessandra Poggi, Francesca Vichi, Elisa Mariabelli, Lorenzo Surace, Giuseppina Berardelli, Orietta Picconi, Alessandra Cenci, Leonardo Sernicola, Claudia Rovetto, Domenico Fulgenzi, Roberto Belli, Emanuela Salvi, Patrizia Di Zeo, Alessandra Borsetti, Barbara Ridolfi, Ruggero Losappio, Fabio Zoboli, Ivan Schietroma, Eleonora Cella, Silvia Angeletti, Massimo Ciccozzi, Stefania D’Amato, Barbara Ensoli, Stefano Buttò, the Italian Network for HIV Characterization, Gioacchino Angarano, Sergio Babudieri, Giuseppe Corano Scheri, Miriam Lichtner, Salvatore Martini, Annamaria Mazzella, Nicola Romano, Alfredo Pansera, Emanuele Pontali, Adriana Raddi, Giulio Starnini, Serena Dell’Isola

**Affiliations:** 1 National Center for the HIV/AIDS Research, Istituto Superiore di Sanità, Rome, Italy; 2 Clinic of Infectious Diseases, Hospital-University Polyclinic, University of Bari, Bari, Italy; 3 Unit of Infectious Diseases, Vittorio Emanuele II Hospital, Bisceglie, Italy; 4 HIV Unit, University of Vanvitelli, Caserta, Italy; 5 Microbiology and Virology Unit, Cotugno Hospital, Naples, Italy; 6 Seroimmunology and Allergology Unit, S. Stefano Hospital, Prato, Italy; 7 Valentini General Medicine Unit, Hospital of Cosenza, Rogliano, Italy; 8 Infectious Diseases Unit, Hospital of Cosenza, Rogliano, Italy; 9 Division of Infectious Diseases, ASST Spedali Civili of Brescia, Brescia, Italy; 10 Department of Public Health and Infectious Diseases, Sapienza University of Rome, Rome, Italy; 11 Department of Molecular Medicine, Sapienza University of Rome, Rome, Italy; 12 Structure of Clinical Pathology, S. Giovanni di Dio Hospital, Florence, Italy; 13 Structure of Infectious Diseases Structure, S.M. Annunziata Hospital, Florence, Italy; 14 Center of Traveller and Migration Medicine, ASP Catanzaro, P.O. Lamezia Terme, Lamezia Terme, Italy; 15 Infectious Diseases Unit, Giovanni Paolo II Hospital, Lamezia Terme, Italy; 16 National Center for Global Health, Istituto Superiore di Sanità, Rome, Italy; 17 Medical Statistic and Epidemiology Research Unit, University Campus Bio-Medico of Rome, Rome, Italy; 18 Unit of Clinical Laboratory Science, University Campus Bio-Medico of Rome, Rome, Italy; 19 Ministry of Health, Directorate General for Prevention, Rome, Italy; 30 Present Address: National Center for drug control and evaluation, Rome, Italy; 31 Present Address: Core Facilities Technical-Scientific Service, Rome, Italy; 32 Present Address: National Center for Research and drug preclinical and clinical evaluation, Rome, Italy; 33 Present Address: Press Office, Istituto Superiore di Sanità, Rome, Italy; 34 Present Address: Department of Clinical and Experimental Medicine, University of Foggia, Foggia, Italy; 20 Clinic of Infectious Diseases, Hospital-University Polyclinic, University of Bari, Bari, Italy; 21 University of Sassari, Sassari, Italy; 22 Department of Public Health and Infectious Diseases, Sapienza University of Rome, Rome, Italy; 23 University la Sapienza Polo Pontino, Rome, Italy; 24 HIV Unit, University of Campania, L.Vanvitelli, Naples, Italy; 25 ASL Napoli 1 Centro, Naples, Italy; 26 Spedali Civili, Brescia, Brescia, Italy; 27 Galliera Hospital, Genoa, Italy; 28 Microbiology and Virology Unit, Cotugno Hospital, Naples, Italy; 29 Belcolle Hospital, ASL Viterbo, Viterbo, Italy

**Keywords:** Infectious diseases, HIV infections, Population screening

## Abstract

The proportion of new diagnoses of HIV infection in immigrants residing in Italy raised from 11% in 1992 to 29.7% in 2018. To investigate the HIV clades circulating in this community a retrospective study was performed in 557 HIV-infected immigrants living in 12 Italian cities. Immigrants originated from East-Europe and Central-Asia (11.7%), North Africa and Middle East (7.3%), South and South-East Asia (7.2%), Latin America and the Caribbean (14.4%), and sub-Saharan Africa (59.4%). More than 87% of immigrants were on antiretroviral therapy (ART), although 26.6% of them were viremic. A 22.0% of immigrants had hepatitis (HBV and/or HCV) and/or tuberculosis. HIV phylogenetic analysis on sequences from 192 immigrants showed the presence of clades B (23.4%), G (16.1%), C (10.4%), A1 (9.4%), F1 (5.2%), D (1.6%) and Circulating Recombinant Forms (CRFs) (33.9%). CRF02_AG represented 72.3% of the total CRFs. Clusters between immigrants and Italian natives were also present. Drug resistance mutations to NRTI, NNRTI, and PI drug classes occurred in 29.1% of ART-treated and in 12.9% of ART-naïve individuals. These data highlight the need for tailored public health interventions in immigrants to avoid spreading in Italy of HIV genetic forms and ART-resistant variants, as well as HIV co-morbidities.

## Introduction

According to the European Centre for Disease, Prevention and Control (ECDC), migrants in the European Union/European Economic Area (EU/EEA) have a higher risk of HIV infection and related co-infections, such as HBV, HCV and tuberculosis (TB), with an increasing trend in populations from Latin America and East Europe, and a decreasing one in populations from sub-Saharan Africa, although they still represent half of diagnosed individuals^1,2^. The number of immigrants resident in Italy has been steadily increasing during the past 8 years and, by the beginning of 2018, more than 5 million foreign individuals were living in Italy (Italian Institute of Statistics, ISTAT, https://www.istat.it/en/). Consequently, the proportion of immigrants with new diagnoses of HIV infection has raised from 11% in 1992 to 29.7% in 2018. Of note, for 72.1% of them the diagnosis was late and less than 6 months prior to developing AIDS^3^. Concerning HIV most frequent co-infections, while the relative proportion of immigrants with chronic HBV infection in Italy has been estimated to be 20%^4^, the relative proportion of HCV infected immigrants is small^5,6^. Finally, a wide study in a cohort of over 27,000 socially marginalized immigrants in Piedmont estimated a prevalence of 2.7% of active TB^7^.

HIV-1 genetic heterogeneity leads to the establishment of an increasing number of subtypes, sub-subtypes and Circulating Recombinant Forms (CRFs), which have a specific geographic distribution that, however, is continuously evolving because of travelling and migration^8^. These genetic forms can be transmitted with different efficiency^9,10^ and have different sensitivity to ART^11^. In addition, HIV diversity can also have an impact on HIV diagnosis and viral load measurement^12^. Thus, public health interventions are needed to limit the spreading of new HIV clades and molecular variants in the population. Indeed, several studies reported that non-B HIV-1 subtypes are now circulating in several previously subtype B-restricted areas of the world, including Italy^13,14^. In particular, the estimated prevalence of infection with non-B subtypes in the Italian population has increased from 2.6% in 1980–1992 to 18.9% in 1993–2008^13^.

A few studies, mainly conducted at a local level, have investigated the heterogeneity of HIV genetic forms in the Italian general population, which also includes migrant communities^15–17^. Our study specifically investigated the distribution of HIV subtypes and CRFs and the presence of their variants carrying mutations of resistance to antiretroviral therapy (ART) in the population of immigrants resident in Italy. In addition, in order to provide more information for health police in these communities, we also described the clinical, virological and immunological characteristics of this population, as well as the presence of HBV and HCV co-infections and TB.

## Results

### Demographic, clinical, immunological and virological data of the study participants

We conducted a retrospective study in Italy using stored plasma samples from 557 HIV-infected resident immigrants, attending clinical centres in the cities of Brescia and Genoa (North Italy); Rome, Florence, Prato, Latina and Viterbo (Centre Italy); Naples, Bari, Cosenza and Lamezia Terme (South Italy); and Sassari (Sardinia) in the period 2008-2017. The demographical, clinical, virological and immunological characteristics of the patients are reported in Table 1. Individuals were native of East Europe and Central Asia (EE & CA, 11.7%), Latin America and the Caribbean (LA & Car, 14.4%), North Africa and Middle East (NA & ME, 7.4%), South and South-East Asia (S & SEA, 7.2%) and Sub-Saharan Africa (SSA, 59.4%).

**Table 1:** Demographical, clinical, immunological and virological characteristics of the study group, stratified by gender.

	Total		Females		Males		p-value
	N	%	n	%	N	%	
**Total**	557	100.0	293	52.6	261*	46.9	—
**Geographical origin**							
EE&CA	65	11.7	35	12.0	30	11.5	p < 0.0001 (χ^2^ test)
LA&Car	80	14.4	23	7.9	55	21.1	
NA&ME	41	7.4	12	4.1	29	11.1	
S&SEA	40	7.2	13	4.4	27	10.3	
SSA	331	59.4	210	71.7	120	46.0	
**Age**							
< = 30	93	16.8	63	21.6	29	11.2	p < 0.0001 (χ^2^ test)
31–45	348	62.7	188	64.4	160	61.5	
46+	114	20.5	41	14.0	71	27.3	
**Co-infections**							
No	379	78.0	201	79.1	177	77.0	p = 0.5630 (χ^2^ test)
Yes	107	22.0	53	20.9	53	23.0	
**Type of co-infection**							
None	379	78.0	201	79.1	177	77.0	p = 0.0320 (χ^2^ test)
HBV	47	9.7	26	10.2	20	8.7	
HCV	31	6.4	12	4.7	19	8.3	
mTB	14	2.9	11	4.3	3	1.3	
Any combination	15	3.1	4	1.6	11	4.8	
Viral Load**							
Undetectable viremia	354	64.6	191	65.6	161	63.4	p = 0.5838 (χ^2^ test)
Detectable viremia	194	35.4	100	34.4	93	36.6	
**Naive to ART with detectable viremia**	**n/N**	**%**	**n/N**	**%**	**n/N**	**%**	
No	125/470	26.6	64/250	25.6	61/218	28.0	p = 0.5613 (χ^2^ test)
Yes	57/64	89.1	32/35	91.4	24/28	85.7	
**Immunological parameters**	**N**	**Mean (SD)**	**N**	**Mean (SD)**	**N**	**Mean (SD)**	
Lymphocytes***	492	1.9 (0.77)	266	1.92 (0.75)	225	2.00 (0.78)	p = 0.2764 Student T-test
**CD4** + ***	508	514.03 (298.61)	271	535.63 (296.95)	235	489.16 (297.33)	p = 0.0800 Student T-test
**CD8** + ***	504	904.59 (481.45)	270	869.13 (453.88)	232	948.25 (509.39)	p = 0.0688 Student T-test
**CD4**+/**CD8**+ **Ratio**	534	0.71 (0.85)	283	0.78 (1.08)	249	0.63 (0.47)	p = 0.0435 Student T-test

*It does not include two transgender patients and one patient whose gender was unknown.

**Detectable viremia was defined as an HIV RNA level ≥ 50 RNA equivalents/ml.

***Cells/μl.

The number of females exceeded that of males (52.6% vs 46.9%). Females were significantly younger than males: 21.6% were less than 30 years of age, as compared to only 11.2% of males (p < 0.0001). One hundred and seven patients out of 486 (22.0%) had at least one co-infection with HBV (9.7%) HCV (6.4%), or *Mycobacterium tuberculosis* (mTB) (2.9%) or a combination of these (3.1%). There was no association between the presence of co-infections and gender. Three hundred-fifty-four people were HIV aviremic (64.6%) and 194 were viremic (35.4%). Patients on ART were 470 (88.0% of 534, i.e. those with available information on ART), while 64 were naive to ART (12.0%), with no differences between males and females. Among those on ART, 125 (26.6%) were still viremic. Finally, females had a statistically significant higher CD4^+^/CD8^+^ ratio than males (p = 0.0435) and also tended to have higher CD4^+^ and lower CD8^+^ T cell numbers than males (p = 0.0800 and 0.0688, respectively).

### HIV subtyping

HIV subtyping was performed on HIV sequences from 192 patients. The distribution of the geographical origins of the HIV-1 subtyped immigrants was similar to that of the total 557 enrolled patients (Supplementary Table S1).

**Figure 1:**
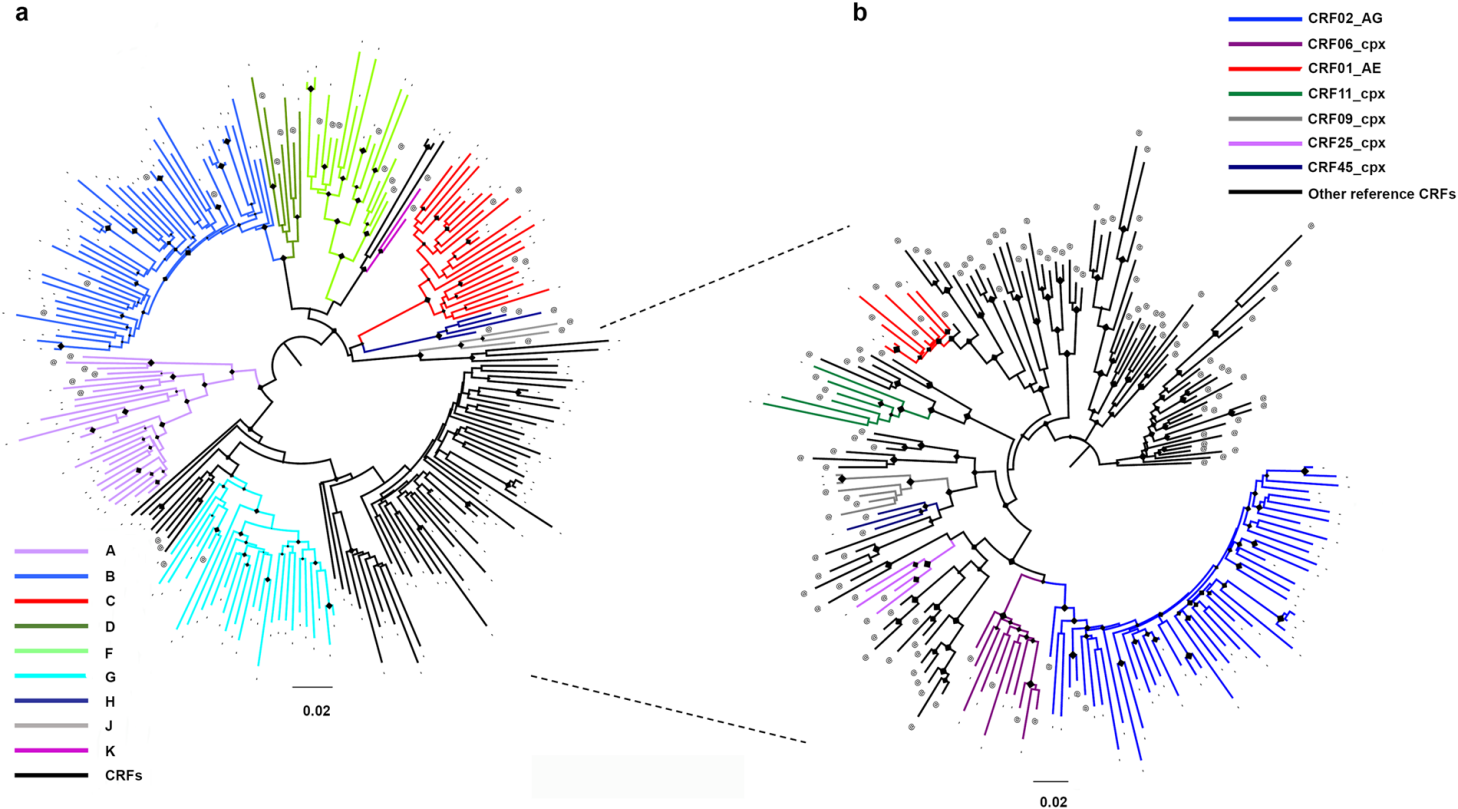
ML phylogenetic tree inferred for HIV-1 genetic forms from 192 HIV-1-infected immigrants. Panel a: ML tree including all the 192 HIV-1 sequences plus pure HIV subtype reference sequences. Panel b: zoom of the ML tree including CRF sequences from our study and CRF reference sequences. “Other CRF reference sequences” are those reference sequences that do not cluster with our sequences. The different subtypes and CRFs are shown in colour, according to the legends present on the top left for panel a, and top right for panel b, respectively. Sequences from our study are indicated with “-“. Reference sequences are indicated with “@”.The diamond (♦) located in the nodes represents significant statistical support for the clade subtending that branch (bootstrap support > 70%). The scale bar indicates 0.02 nucleotide sequence difference. Frequency of each pure subtype and CRF is shown at the bottom of panel a and b, respectively.

Figure 1 reports the phylogenetic relationships among these sequences, using Maximum Likelihood (ML) trees. Many statistically supported clusters were found, indicating the presence of different pure subtypes and CRFs. Forty-five HIV sequences were identified as pure B-subtypes, 82 as pure non-B subtypes and the remaining 65 as probable CRFs (Fig. 1, panel a). Analysis of the CRF pool was further expanded and identified 7 different CRF and cpx sequences (Fig. 1, panel b). These results are in line with those obtained using the REGA subtyping tool.

**Figure 2:**
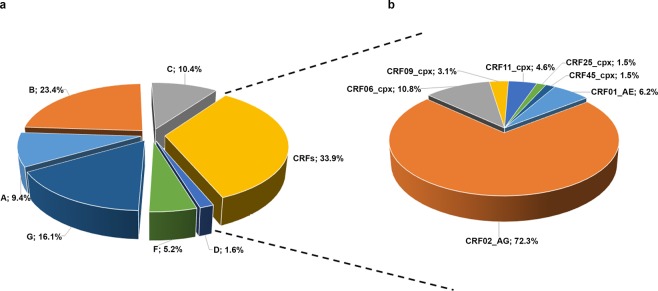
Prevalence of HIV-1 subtypes and recombinant forms in 192 immigrants resident in Italy. The prevalence of the genetic forms is expressed as the percentage of the total number.

The prevalence of HIV-1 subtypes and CRFs in the 192 immigrants is shown in Fig. 2. Overall, the 192 patients were infected by a wide diversity of subtypes and recombinant forms. Subtype B represented 23.4% of the infecting HIV-1 viruses. The remaining non-B subtypes were detected in 76.6% of the patients and they included subtypes G (16.1%), C (10.4%), A1 (9.4%), F1 (5.2%) and D (1.6%). CRFs accounted for 33.9% of the total genetic forms (Fig. 2, panel a). CRF02_AG represented 72.3% of the total CRFs, followed by CRF06_cpx (10.8%), CRF01_AE (6.2%), CRF11_cpx (4.6%), CRF09_cpx (3.1%), CRF25_cpx (1.5%) and CRF45_cpx (1.5%) (Fig. 2, panel b).

### Distribution of HIV-1 genetic forms according to the geographical origin

**Figure 3:**
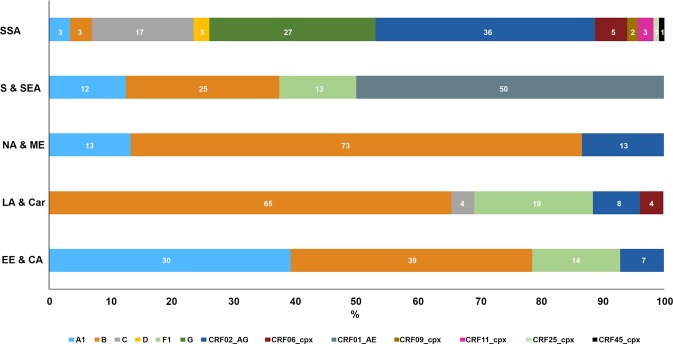
Distribution of HIV-1 subtypes and recombinant forms by geographical area of origin of 192 immigrants resident in Italy. Each subtype and CRF is identified by a colour according to the legend reported at the bottom of the figure. The prevalence of each genetic form is reported as the percentage of the total number for each region.

The distribution of the HIV-1 genetic forms according to the geographical origin of immigrants is shown in Fig. 3. Almost all of them were present in individuals from SSA, in particular all CRFs, with the only exception of CRF01_AE (dark grey colour), circulating exclusively in individuals from S&SEA (50% of the S&SEA sequences). The B subtype (orange colour) was present in patients from all the geographical regions, with a prevalence varying from 73% and 65% for NA&ME and LA&Car, respectively, to 3% for SSA. Individuals from SSA were also the only ones infected by subtype D (ochre yellow) and G (dark green). Subtype C strains (light grey) were present in patients from SSA and LA&Car with a frequency of 17% and 4%, respectively. CRF02_AG (deep blue colour) and the A1 subtype (light blue colour) were present, with various prevalence, in patients from all the regions except S&SEA (CRF02_AG) and LA&Car (A1). The F1 subtype (light green) was present in patients from S&SEA (13%), LA&Car (19%) and EE&CA (14%), and the recombinant form CRF06_cpx (dark red) was present in patients from SSA and LA&Car (5% and 4%, respectively). Finally, CRF09_cpx, CRF11_cpx, CRF25_cpx and CRF45_cpx were present with a low frequency exclusively in patients from SSA.

### Differences among clades for the immunological and virological parameters

We then evaluated if there were differences in the immunological and virological parameters (absolute number and percentage of lymphocytes; CD4 and CD8; CD4/CD8 ratio; HIV viral load) according to clades. When ART-naïve patients were considered, no statistically significant differences among HIV-1 clades were observed (p > 0.05 by Kruskal-Wallis test). However, in ART-treated patients, the CD4 percentage and the CD4/CD8 ratio differed among clades (0.0007 and 0.004, respectively, by Kruskal-Wallis test). In particular, a statistically significant difference was found for subtype A1 vs CRF, subtype B vs CRF and subtype C vs subtype G for the CD4 percentage (Wilcoxon-Mann-Whitney test p = 0.011, p = 0.0054 and p = 0.0495, respectively) and for subtype A vs CRF and subtype C vs G for the CD4/CD8 ratio (p = 0.0271 and p = 0.0436, respectively) (Supplementary Table S2).

### Clustering analysis

Behaviours at-risk for HIV infection were known for 119 immigrants. Supplementary Table S3 reports the behaviours of these immigrants, as compared to those of 136 Italian autochthonous individuals, living in the cities of Brescia, Genoa, Sassari, Naples and Bari, whose samples were available at the centres. The most common HIV risk behaviours were unprotected homosexual (Men who have Sex with Men, MSM) and heterosexual intercourses in natives (41.9% and 32.4%, respectively) and unprotected heterosexual intercourses in immigrants (58.8%).

**Figure 4:**
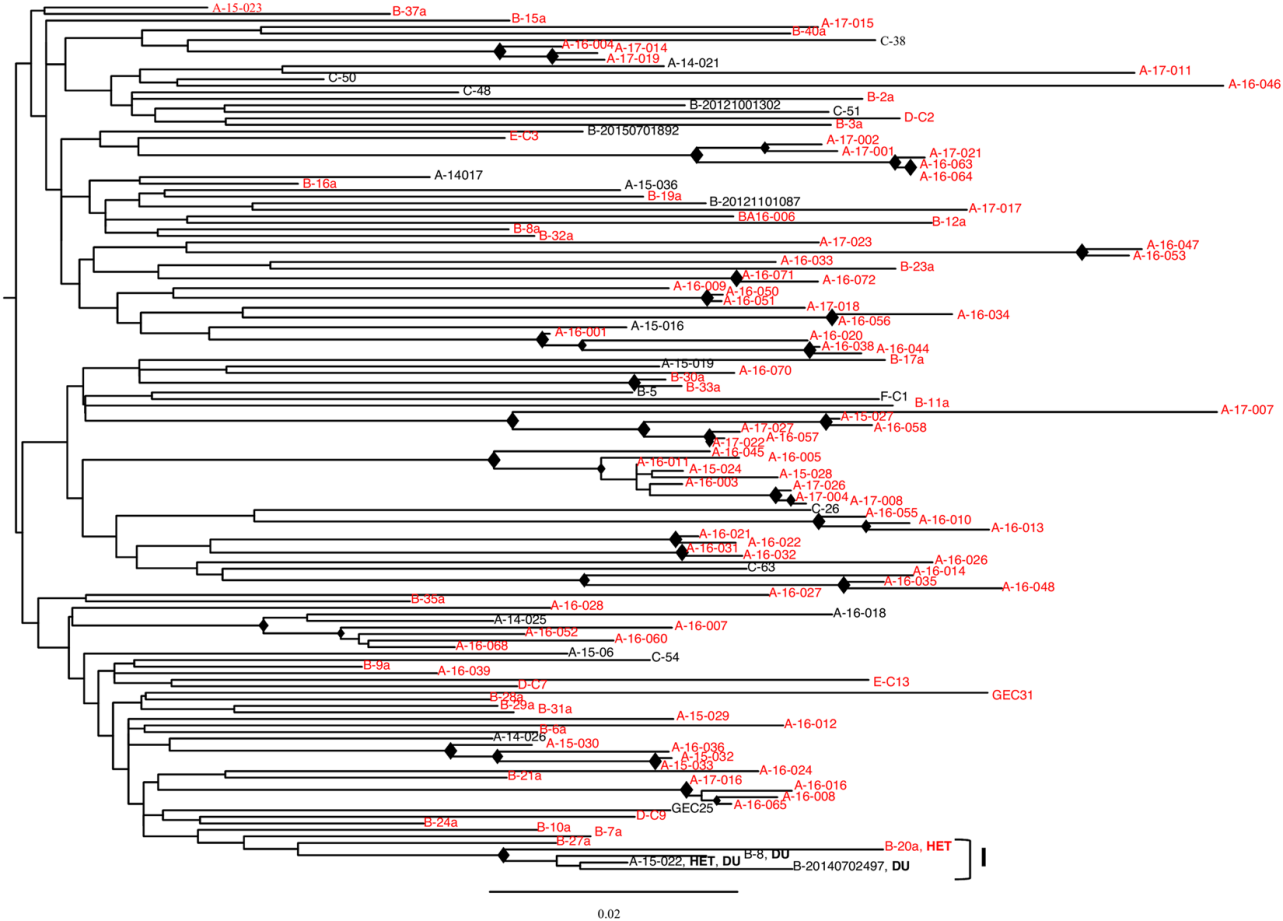
Clustering analysis of HIV-1 sequences from 108 Italians and 25 immigrants infected by HIV-1 subtype B, according to HIV risk behaviour and demographical data. ML trees are shown. The diamond (♦) located in the nodes represents significant statistical support for the clade subtending that branch (bootstrap support > 70%). Roman numbers indicate clusters. Names include the city one-letter code, and an internal patient code. The risk factor (DU: Drug User; HET: Heterosexual) is reported only for patients included in a cluster. Italian natives are indicated in red, immigrants in black. The scale bar indicates 0.02 nucleotide sequence difference.

**Figure 5:**
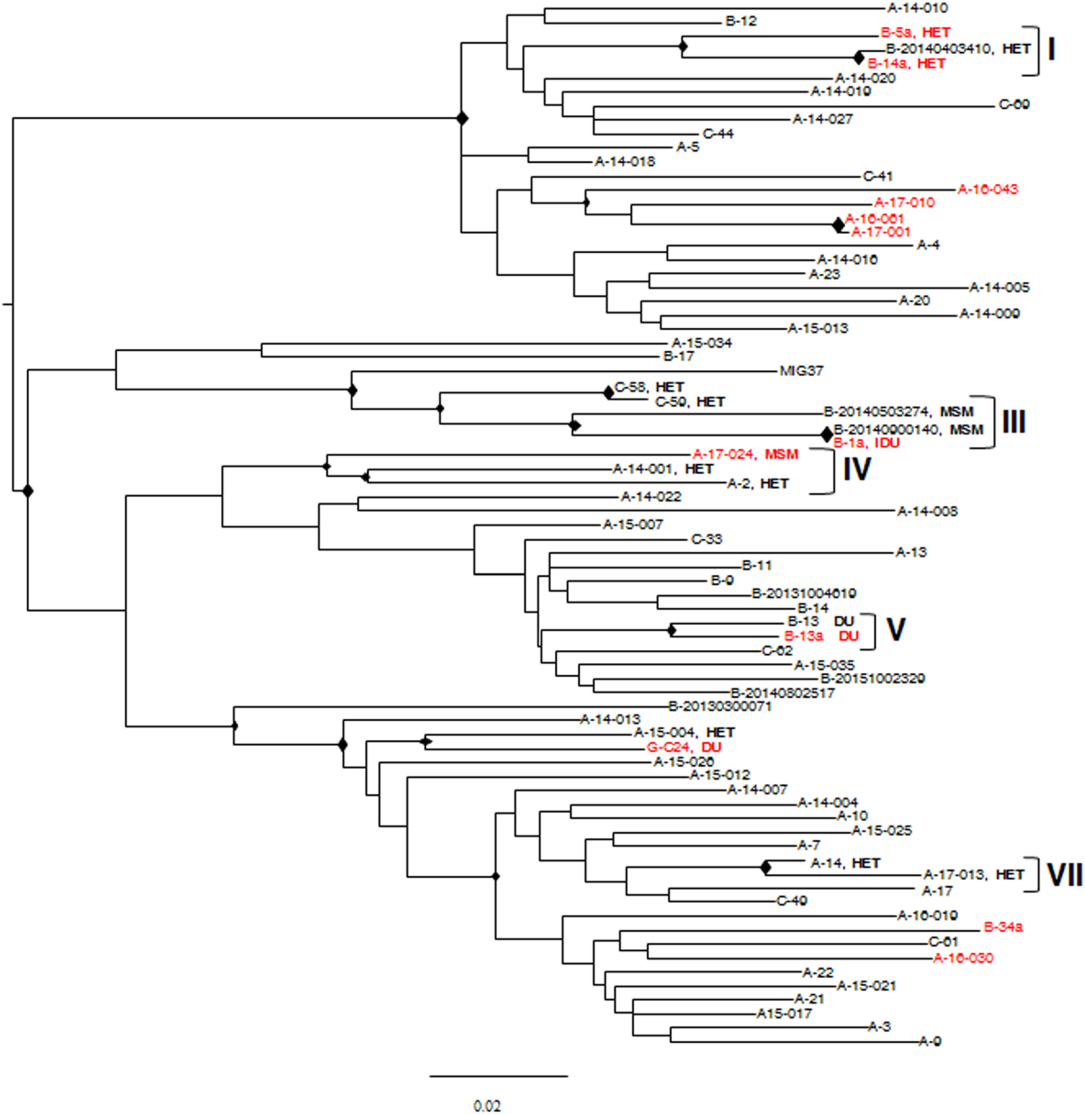
Clustering analysis of HIV-1 sequences from 12 Italians and 63 immigrants infected by HIV-1 non-B subtypes, according to HIV risk behaviour and demographical data. ML trees are shown. The diamond (♦) located in the nodes represents significant statistical support for the clade subtending that branch (bootstrap support > 70%). Roman numbers indicate clusters. Names in the cluster include the city one-letter code and an internal patient code. The risk factor (DU: Drug User; HET: Heterosexual; MSM: Male-to-Male Sex) is reported only for patients included in a cluster. Italian natives are indicated in red, immigrants in black. The scale bar indicates 0.02 nucleotide sequence difference.

**Figure 6:**
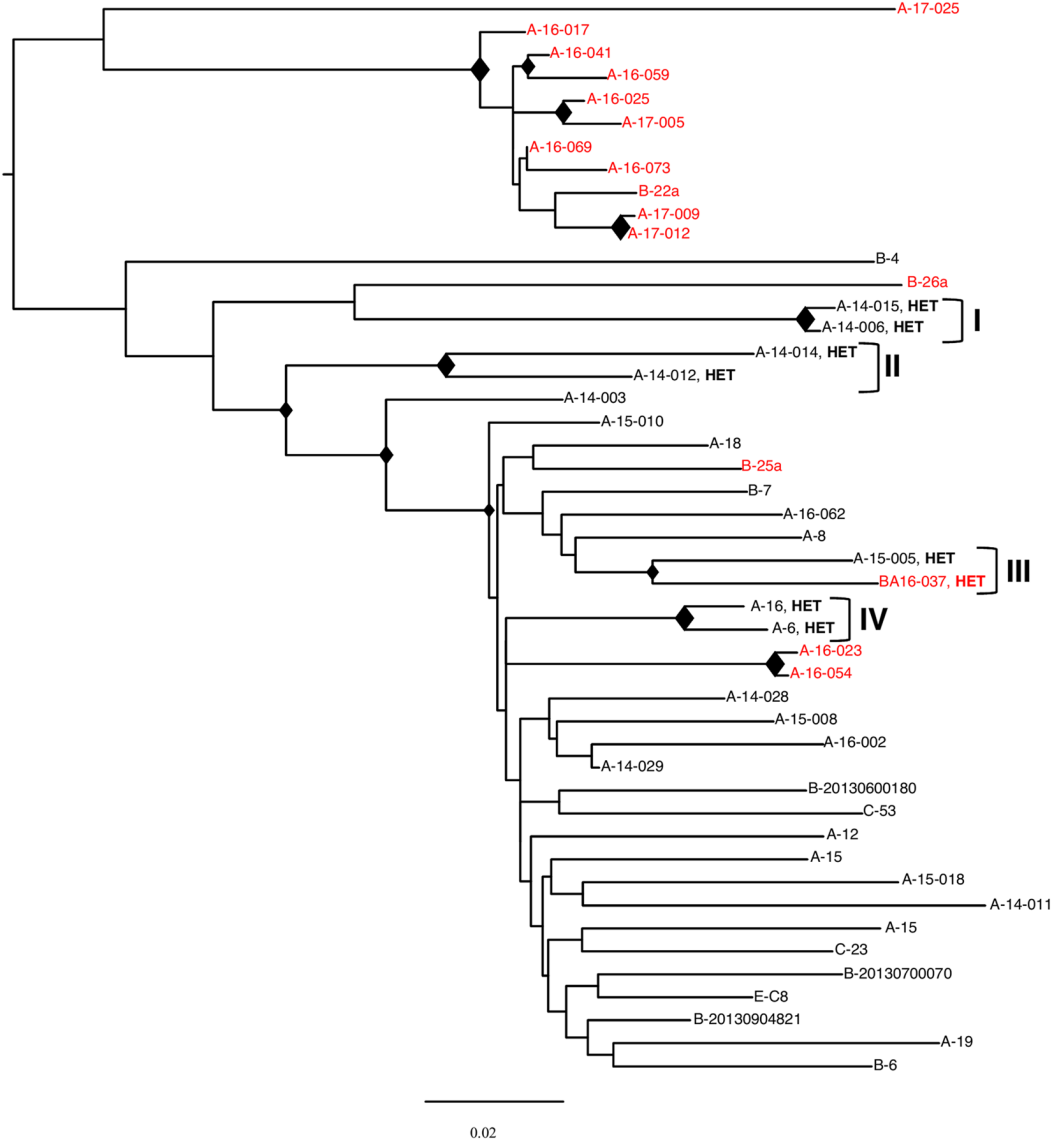
Clustering analysis of HIV-1 sequences from 16 Italians and 31 immigrants infected by HIV-1 CRFs, according to HIV risk behaviour and demographical data. ML trees are shown. The diamond (♦) located in the nodes represents significant statistical support for the clade subtending that branch (bootstrap support > 70%). Roman numbers indicate clusters. Names in the cluster include the city one-letter code and an internal patient code. The risk factor (HET: Heterosexual) is reported only for patients included in a cluster. Italian natives are indicated in red, immigrants in black. The scale bar indicates 0.02 nucleotide sequence difference.

In order to study the transmission between imported and local HIV variants, sequences from the 119 immigrants and the 136 Italians were analysed by subtype and according to the HIV risk behaviour and demographic data (Figs. 4, 5 and 6).

Figure 4 reports the ML tree of subtype B sequences. There is only one statistically supported cluster (bootstrap value > 0.7), including 4 sequences from male patients, 2 from drug abuser immigrants, one from an immigrant who reported both drug use and unprotected heterosexual contacts, and a fourth one from an autochthonous heterosexual patient. Three patients (two drug users from different countries and the heterosexual Italian native) were resident in the same city.

Figure 5 reports the ML tree of pure non-B sequences. Seven clusters were identified. Cluster I includes C subtype sequences and is composed of three heterosexual patients, living in the same city, two autochthonous and one from Colombia. Clusters II and III include patients infected by F subtype strains. Cluster II is composed of two sequences from Romanian heterosexual patients, one male and one female resident in the same city, whereas cluster III consists of three sequences from patients living in the same city, two male Romanian homosexual patients and one male autochthonous drug abuser. Clusters IV and V include subtype A1 infected patients. Cluster IV is composed of two heterosexual patients, one male and one female from Pakistan and Eritrea, respectively, and a male autochthonous homosexual patient, all living in the same city. Cluster V consists of two sequences isolated from two drug abusers from the same city, one autochthonous and one from Tunisia. Finally, clusters VI and VII include sequences from the G subtype. Clusters VI is composed of a heterosexual patient from Cameroon and an autochthonous drug abuser, whereas cluster VII of two heterosexual patients from Nigeria, one male and one female, resident in the same city.

Figure 6 reports the ML tree including sequences from patients infected by HIV-1 CRFs. Four statistically supported clusters were found. Only cluster III shows an intermixing between immigrant and autochthonous sequences and includes two sequences from heterosexual patients, an autochthon and a patient from Ukraine. The remaining clusters are all sequences from immigrants. Cluster I consists of a heterosexual patient and a patient reporting having been poly-transfused in the country of origin. Both patients were resident in the same city, native of the Ivory Coast and infected by the HIV-1 genetic form CRF09_cpx. Cluster II includes two heterosexual patients, a male from Liberia and a female from Burkina Faso, both infected by the HIV-1 CRF06_cpx. Finally, cluster IV is composed of two heterosexual individuals, a male and a female, both from Nigeria and infected by the HIV-1 CRF02_AG.

### ART resistance mutations

Overall, 45 out of 192 (23.4%) immigrants hosted HIV-1 variants carrying at least one major Drug Resistance Mutation **(**DRM) to one class of drugs (Table 2). Of these, 37 were on ART, 7 were ART naïve while for 1 person no information on the therapy was available. The 37 patients on ART with DRMs represented 29.1% of the ART-treated patients (37/127). The most common DRMs were M184V (present in 8 patients) for NRTIs, and K103N (present in 10 patients) for NNRTIs, respectively. Thirteen mutations were also present for PIs, which included drugs with a high genetic barrier such as darunavir and lopinavir. Among the 54 ART-naïve patients, 7 (12.9%) hosted HIV variants with DRMs. In particular, 5 patients had DRMs directed to NNRTIs (E138K, G190A, V108I, V179E, V179F), 1 to NRTIs (D67N) and 1 to PIs (L90M), respectively.

**Table 2:** Major drug resistance mutations (DRMs) in 192 migrants resident in Italy characterized for the infecting HIV-1 clade.

HIV-1 subtyped patients				Major DRMs		
*ART status*	*Total*	*Without DRMs*	*With DRMs*	*NRTI*	*NNRTI*	*PI*
ART naïve	54	47	7	D67N (1)*	V108I (1); E138K (1); V179E (1); V179F (1); G190A (1)	L90M (1)
On ART	127	90	37	M184V (8); A62V (2); M41L (1); K65R (1); D67S (1); T69G (1); L74I (1); L74V (1); M184I (1); T215V (1)	K103N (10); Y181C (4); V179E (3); A98G (2); V108I (2); E138A (2); V179D (2); Y188L (2); K101E (1); K103T (1); V106I (1); Y181H (1); Y188F (1); Y188H (1); H221Y (1); M230I (1); Y318F (1);	M46I (3); I54V (2); V82A (2); V32I (1); I47V (1); I50V (1); I84V (1); N88G (1)
Unknown	11	10	1	A62V (1)	No mutations	No mutations
TOTAL	192	147	45			

*In parenthesis the number of patients carrying variants with that specific mutation.

**Figure 7:**
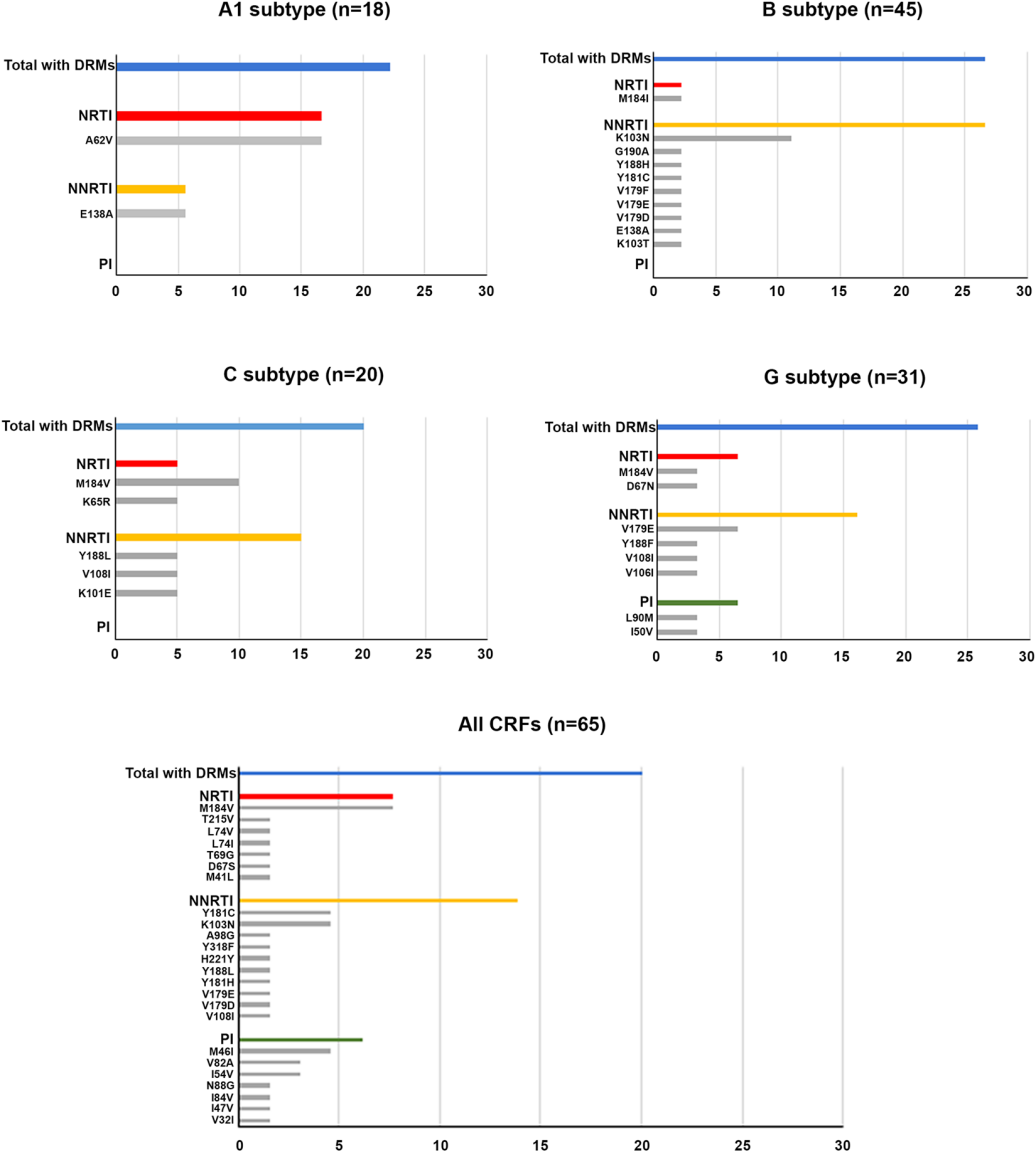
Relative frequency of patients with DRMs according to the genotype. The relative frequency of patients with DRMs is indicated for subtypes A1, B, C, G and for all CRFs. The number of sequences included in the analysis is indicated in brackets at the top of each clade. Blue bar: total frequency of DRMs; red bar: frequency of DRMs to NRTIs; yellow bar: frequency of DRMs to NNRTIs; green bar: frequency of DRMs to PIs; grey bar: frequency of each single mutation.

We then investigated the frequency of patients carrying the major DRMs according to the HIV-1 clades (Fig. 7). All subtypes and the CRF02_AG were composed of 18 or more patients, while subtypes D and F1 included only 3 and 10 patients, respectively, and CRF01_AE, CRF06_cpx, CRF09_cpx, CRF11_cpx, CRF25_cpx and CRF45_cpx included a few patients each (ranging from 1 patient for CRF25_cpx and CRF45_cpx to 10 patients for CRF09_cpx). Therefore, due to the low number, the D and F1 subtypes were not included in the analysis and all the CRFs were merged together to increase the statistical power of the analysis. Overall, A1, B, C and G pure subtypes and CRFs showed a frequency of patients carrying DRMs between 20.0% and 26.7%, with the B subtype having the highest frequency, and the CRFs and subtype C the lowest one. However, this difference is not statistically significant (χ^2^ > 0.05). When each drug class was considered, only the G subtype, among the pure subtypes, and the CRFs showed mutations against all the NRTI, NNRTI and PI drug classes. Mutations against NNRTIs were the most frequent ones in all clades, except for the A1 subtype that showed a higher frequency of mutations to NRTIs. When considering each mutation singularly, CRFs and subtype B showed the highest number of different mutations, but due to the low frequency of each mutation, a statistical treatment was not carried out.

## Discussion

Our paper describes the HIV clade heterogeneity in HIV resident immigrants in Italy. Previous studies were conducted in a few areas providing only a partial picture of the dynamics of the HIV genetic forms circulating in the country^13,15,18^. The present study also describes the immunological and virological characteristics of the study subjects and related co-infections, with the aim of providing more clinical information on the immigrants resident in Italy. However, being a retrospective study some information is missing, such as, for example, the possibility of determining where the patient acquired the infection (in the country of origin, in Italy or elsewhere).

In our study, females were more numerous and significantly younger than males. This is not surprising since more than half of patients were from SSA, where HIV is transmitted mainly by unprotected heterosexual intercourses and women are disproportionately affected by HIV, accounting for approximately 58% of all people living with HIV-1 in the region^19^. Similarly, SSA women tended to be infected at a younger age than men, and about 75% of new infections occurred in 15–19 years old women^20^ and https://www.unaids.org/sites/default/files/women_girls_hiv_sub_saharan_africa_en.pdf.

Women had better immune-virological parameters than males. This is in line with previous data indicating that women on ART show a consistently better CD4^+^ T cell recovery than men^21^, although in some cases they may have greater levels of immune activation and inflammatory activity and a faster progression to AIDS than men^22^.

Only 73.8% of immigrants on ART was found to control viremia. This is in agreement with previous data in ART-treated migrants in Italy showing that only 77.7% of them were aviremic, as opposed to 84.2% of Italian native patients^23^. This difference could be explained by a greater difficulty of migrants to access health care and, consequently, by a lower compliance to therapy, and it is in line with data confirming an increased risk of virological failure to the first antiretroviral regimen in HIV-infected migrants as compared to Italian natives^24^. However, the role of the infecting clade cannot be ruled out.

In our study, 22.0% of the total immigrants had at least one co-infection with HBV and/or HCV and/or had TB. In particular, 9.7% was co-infected with HBV, 6.4% with HCV and 2.9% with *m*TB. The 2016 ECDC Technical Report on the “Epidemiological assessment of hepatitis B and C among migrants in the EU/EEA” indicates a range of estimated prevalence of active HBV infection among migrants from intermediate and high-endemic countries from 3% (Estonia, Latvia, Lithuania and Poland) to 9% in Portugal; and a prevalence of viremic HCV infection ranging from 0.9% in Croatia to 2.4% in Latvia^25^, respectively. In addition, a recent study shows that in a population of newly arrived migrants in Modena (Italy), the prevalence of active HBV and HCV infections and active TB is 12.2%, 3.3% and 2.0%, respectively^6^. The prevalence data from our study are slightly different, possibly due to a different composition of the migrant communities present in our study.

Phylogenetic analysis of HIV genetic forms revealed that 76.6% of the subtyped immigrants were infected with non-B subtype strains. These data are remarkably different from those found in the general population in Italy, which indicate an 18.9% prevalence of non-B subtypes, although this prevalence is increasing over time^13^.

In our study, the recombinant forms were present in 33.9% of immigrants, with CRF02_AG representing 72.3% of the total CRFs. CRF02_AG has been estimated to be responsible for at least 8% of the infections worldwide^26^. Although, the CRFs distribution varies according to the different geographical regions, the prevalence of CRFs has been described to increase overtime, both globally and regionally, including in Italy^18,26^.

We found the CRF06_cpx in patients from Nigeria, Senegal, Burkina Faso, Liberia and Cuba. To our knowledge, the presence of CRF06_cpx was not previously described in Cuba. In our study, the Cuban patient was a 27-year old woman. No information on risk behavior for HIV infection and the year of entry in Italy was reported for this patient. Thus, based on the available information, we cannot rule out the possibility that the patient acquired this HIV genetic form outside of her country of origin, in Italy, or elsewhere.

The genetic forms isolated from immigrants were, overall, in agreement with their presence in the countries of origin, although the prevalence of each genetic form by geographical area often does not reflect what is reported in the literature^27,28^. This may depend on the distribution of the communities of immigrants in our country and in our study group. For example, the unusual high prevalence of the F1 subtype (14%) found in immigrants from EE&CA is due to the presence of many immigrants from Romania, who are the largest foreign community in Italy. In Romania, the HIV-1 epidemic has been described to be unique, as the globally rare subtype F1 predominates with a 95% prevalence^27,29,30^.

We found a statistically significant difference in some immunological parameters (CD4 percentage and CD4/CD8 ratio) among clades in ART-treated patients. In particular, a statistically significant difference was found between subtype A vs CRF and subtype C vs G for the CD4/CD8 ratio, with subtypes A and C showing a higher CD4/CD8 ratio. A low CD4/CD8 ratio has been found to be associated with increased risk of serious events and deaths in ART-treated patients, even in the presence of viral suppression^31^. However, our data are not sufficient to draw any conclusion on the association between specific genetic forms and a low CD4/CD8 ratio.

Clustering analysis of pure non-B and CRF isolates showed the presence of clusters between immigrants and Italian natives. No clusters were present among HIV-1 B subtype-infected immigrants and only a cluster between immigrants and autochthonous sequences were found. This is consistent with the higher prevalence of pure B subtype in the Italian autochthonous individuals, as compared to the immigrant population^13^. Finally, the clustering analysis of non-B pure subtype and CRF sequences indicated that the unprotected heterosexual contacts are a main route of transmission both among immigrants and between immigrants and Italian natives. This is in line with the ECDC HIV/AIDS surveillance data in Europe (2018), indicating that 40% of newly diagnosed cases in the EU/EEA area that are due to heterosexual transmission are among migrants^32^. This identifies the migrant population as a key vulnerable population requiring specific prevention and control measures in EU/EEA countries.

We detected DRMs in 23.4% (45/192) of patients. Among ART-treated people, 29.1% of patients (37/127) carried variants with major DRMs. The most common mutations were K103N (13.3% of all mutations) and M184V (10.6% of all mutations) that confer resistance to NNRTI and NRTI, respectively. The M184V mutation has been suggested to be a marker of noncompliance to therapy^33^. We also found DRMs in 7 out of 54 ART-naïve patients (12.9%), indicating that these mutations have been transmitted (Transmitted Drug Resistance Mutations, TDRMs). This is in line with data from the literature, reporting that the prevalence of TDRMs varies from 0% to 15%, rarely exceeding 20%, depending on the geographical area, HIV prevalence, routes of transmission and infecting subtype^34–37^. Five out of the 7 patients with TDRMs had mutations conferring resistance to NNRTI drugs. This is in line with data indicating a higher prevalence of TDRMs to NNRTIs (for a complete review on drug resistance, globally, see the WHO HIV drug resistance report 2017 at https://www.who.int/hiv/pub/drugresistance/hivdr-report-2017/en/).

Frequency of mutations was similar among HIV clades, ranging from 20% to 26.7%. This suggests that drug resistance affects to a similar extent all HIV-1 clades, as recently described^38^. The most represented mutations in all clades were those of the NNRTI drug class, in agreement with data reported in the literature^39–41^, indicating that mutations to NNRTIs tend to increase overtime. On the contrary, subtype A showed a greater prevalence of mutations to NRTI drug class. However, the limited number of subtype A-infected patients in our study does not allow drawing any firm conclusions on the possible relation between subtype A and mutations to NRTI drug class.

The main findings from our study are the presence of a high variability of HIV genetic forms and the presence of ART-resistant HIV variants in the immigrant population resident in Italy, including ART naïve individuals. This indicates that a continuous surveillance of HIV genetic forms is needed in Italy, and that this should be accompanied by specific public health interventions targeting the immigrant community in order to limit spreading of different HIV genetic forms both in the immigrant community and in the general population in Italy.

## Methods

### Patients

This retrospective cross-sectional study was carried out using stored plasma samples from 557 HIV-positive adult immigrants resident in Italy in the period 2008–2017, who were attending clinical centres of the North (Brescia and Genoa), Centre (Rome, Florence, Prato, Latina and Viterbo), South (Naples, Bari, Cosenza and Lamezia Terme) Italy, and Sardinia (Sassari). Demographic, behavioural, clinical, immunological and virological data were obtained from the clinical centres. Plasma samples were collected in 2008 (1 patient), 2009 (8), 2010 (90), 2011 (50), 2012 (56), 2013 (241), 2014 (31), 2015 (54), 2016 (25) and 2017 (1). Diagnosis of HBV and HCV infection and TB was performed at the clinical centres.

### Ethical statement

The Ethical Committee of ISS approved the study (Prot. PRE 1115/18, March 16, 2018). Being a retrospective study, the Ethics committee has authorized its conduct without the need to obtain a specific informed consent from the participants, because the study falls under the conditions foreseen in the authorization no. 9/2016 - General Authorization to Process Personal Data for Scientific Research Purposes of the Italian Data Protection Authority.

Data were processed using unique identifiers to ensure confidentiality. The study and the treatment of personal data were conducted according to the Italian law 196/2003 and the EU regulation of the European Parliament and the European Council n. 2016/679.

### HIV subtyping and clustering analysis

HIV subtyping from plasma was successful in 192 patients with detectable viremia. Plasma samples from these patients were collected in 2009 (4 patients), 2010 (43), 2011 (23), 2012 (24), 2013 (17), 2014, (29), 2015 (36), 2016 (15), 2017 (1).

The PR-RT region of the HIV-1 genome was amplified and sequenced, as previously described^42^. Sequences are registered in Genbank (Genbank accession no. from MN133054 to MN133227 and from MN165019 to MN165036).

The phylogenetic relationships (HIV subtyping and clustering) among the HIV-1 PR-RT sequences were investigated by building five datasets. The first dataset included the 192 PR-RT sequences from the patients and 39 M group pure subtype reference sequences (A to K), downloaded from the National Center for Biotechnology Information (NCBI) (https://www.ncbi.nlm.nih.gov/). The 39 reference sequences included: subtype A (6 sequences), B (4 sequences), C (4 sequences), D (4 sequences), F (8 sequences), G (4 sequences), H (4 sequences), J (3 sequences) and K (2 sequences). The second dataset included only sequences from patients classified as probable CRFs (65 PR-RT sequences), using the first dataset plus 119 CRF reference sequences from NCBI. These two datasets were used for HIV subtyping. In order to infer the phylogenetic relationship among strains based on the risk factors, HIV clade and demographic data, other three datasets (third, fourth and fifth dataset) were built, including only sequences from patients for whom demographic and epidemiological and behavioural information was available. Sequences from both HIV-infected immigrants (119) and HIV-infected Italian natives (136) were included. Italian patients were enrolled in the period 2011–2017 in the clinical centres of Brescia, Genoa, Sassari, Naples and Bari. The third dataset was composed of 25 PR-RT sequences from immigrant patients, and 108 from Italian natives, all infected by subtype-B. The fourth dataset was composed of 75 PR-RT sequences from immigrant patients and 12 from Italian natives, all infected by pure non-B subtypes. The fifth dataset included 47 PR-RT sequences from immigrant patients and 16 from Italian natives, all infected by probable CRFs.

All sequences were aligned using MAFFT^43^ and manually edited with Bioedit^44^, removing gaps and cutting to identical sequence lengths. The viral subtype was first determined with the REGA HIV-1 Subtyping Tool (version 3.0 http://dbpartners.stanford.edu:8080/RegaSubtyping/stanford-hiv/typingtool/) and confirmed by phylogenetic analysis through ML phylogenetic tree using the first and second dataset. MEGA7^45^ was used to select the simplest evolutionary model that adequately fitted the sequence data for the datasets by using the “Models” tool. The statistical robustness and reliability of the branching order within the phylogenetic tree was confirmed by the bootstrap analysis (bootstrap values > 70% identified statistically supported clusters of sequences).

### Identification of drug resistance mutations

The presence of major DRMs was investigated in all subtyped patients, according to the WHO-2009 list of resistance mutations^46^, the 2019 updated IAS-USA Drug resistance mutations in HIV-1 list^47^ and the Stanford HIV Drug Resistance database^48^, last updated on 2019-07-10. Only major resistance mutations were considered in the analysis. Accessory mutations were excluded.

### Statistical analysis

Descriptive statistics summarizing quantitative variables included mean, standard deviation, standard error, median, 25^th^ and 75^th^ percentiles. Frequency distributions were presented for categorical variables. Comparison between categories were performed using the Chi-Square or Fisher’s Exact Test, while Student T-test was used in order to compare quantitative variables.

Comparisons of continuous variables among different groups was performed by using nonparametric tests (Kruskal-Wallis test, Mann-Whitney test). Statistical analyses were carried out at two-sided with a 0.05 significance level, using SAS^®^ (Version 9.4, SAS Institute Inc., Cary, NC, USA).

## Supplementary information

Supplementary information.
