# Supplementary material for: High HIV-1 diversity in immigrants resident in Italy (2008–2017)
Source: Sci Rep. 2020 Feb 24;10:3226. doi: 10.1038/s41598-020-59084-2 (PMC7039940; doi:10.1038/s41598-020-59084-2)
Supplement: Supplementary file 1 — Supplementary information. [file 41598_2020_59084_MOESM1_ESM.pdf]

High HIV-1 diversity in immigrants resident in Italy (2008-2017)

Maria Teresa Maggiorella[1], Nunzia Sanarico [1]*^, Gaetano Brindicci[2], Laura Monno[2], Carmen Rita Santoro[3], Nicola Coppola[4], Nunzia Cuomo[5], Annalisa Azzurri[6], Francesco Cesario[7], Filippo Luciani[8], Issa El-Hamad[9], Gabriella D'Ettorre[10], Ombretta Turriziani[11], Laura Mazzuti[11], Alessandra Poggi[12], Francesca Vichi[13], Elisa Mariabelli[13], Lorenzo Surace[14], Giuseppina Berardelli[15], Orietta Picconi[1], Alessandra Cenci[1#], Leonardo Sernicola[1], Claudia Rovetto[1], Domenico Fulgenzi[1], Roberto Belli[1], Emanuela Salvi[1§], Patrizia Di Zeo[1@], Alessandra Borsetti[1], Barbara Ridolfi[16], Ruggero Losappio[3], Fabio Zoboli[9$], Ivan Schietroma[10], Eleonora Cella[17], Silvia Angeletti[18], Massimo Ciccozzi[17], Stefania D'Amato[19], Barbara Ensoli[1] Stefano Buttò[1]**, and the Italian Network for HIV Characterization.*

[1]National Center for the HIV/AIDS Research, Istituto Superiore di Sanità, Rome, Italy; [2]Clinic of Infectious Diseases, Hospital-University Polyclinic, University of Bari, Bari, Italy; [3]Unit of Infectious Diseases, Vittorio Emanuele II Hospital, Bisceglie, Italy; [4]HIV Unit, University of Vanvitelli, Caserta, Italy; [5]Microbiology and Virology Unit, Cotugno Hospital, Naples, Italy;[6]Seroimmunology and Allergology Unit, S. Stefano Hospital, Prato, Italy; [7]Valentini General Medicine Unit, Hospital of Cosenza, Rogliano, Italy; [8]Infectious Diseases Unit, Hospital of Cosenza, Rogliano, Italy; [9]Division of Infectious Diseases, ASST Spedali Civili of Brescia, Brescia, Italy; [10]Department of Public Health and Infectious Diseases, Sapienza University of Rome, Rome, Italy; [11]Department of Molecular Medicine, Sapienza University of Rome, Rome, Italy; [12]Structure of Clinical Pathology, S. Giovanni di Dio Hospital, Florence, Italy; [13]Structure of Infectious Diseases Structure, S.M. Annunziata Hospital, Florence, Italy; [14]Center of Traveller and Migration Medicine, ASP Catanzaro, P.O. Lamezia Terme, Lamezia Terme, Italy; [15]Infectious Diseases Unit, Giovanni Paolo II Hospital, Lamezia Terme, Italy; [16]National Center for Global Health, Istituto Superiore di Sanità, Rome, Italy; [17]Medical Statistic and Epidemiology Research Unit, University

Campus Bio-Medico of Rome, Rome, Italy; [18]Unit of Clinical Laboratory Science, University Campus Bio-Medico of Rome, Rome, Italy; [19]Ministry of Health, Directorate General for Prevention, Rome, Italy.

Present addresses:

^National Center for drug control and evaluation, #Core Facilities Technical-Scientific Service, §National Center for Research and drug preclinical and clinical evaluation, @Press Office, Istituto Superiore di Sanità, Rome, Italy, $Department of Clinical and Experimental Medicine, University of Foggia, Foggia, Italy.

*Equal contributors

Supplementary Table S1. Geographical origin of total and subtyped migrants

	Total number		Number of subtyped	
**Geographical area of origin**	***n*[a]**	**%**	***n*[b]**	**%**
EE & CA	65	11.7	28	14.6
LA & Car	80	14.4	26	13.5
NA & ME	41	7.3	15	7.8
S & SEA	40	7.2	8	4.2
SSA	331	59.4	115	59.9

a = Total: 557 individuals
b = Total: 192 individuals

Supplementary Table S2. Median of CD4 percentage and CD4/CD8 ratio among HIV-1 clades

**Parameter**	**Clades**					
	**A**	**CRF**	**B**	**CRF**	**C**	**G**
**CD4%**	28.00	14.79	21.52	14.79	28.29	18.00
	p*=0.0011		p*=0.0054		p*=0.0495	
**CD4/CD8**	0.60	0.30	0.40	0.30	0.59	0.33
	p*=0.0271		p*>0.05		p*=0.0436	

* Wilcoxon-Mann-Whitney test

Supplementary Table S3. Behaviours at-risk of HIV infection in Italian and immigrant HIV-infected patients

At risk behaviour	Number	
	**ITALIANS (% on total)**	**IMMIGRANTS (% on total)**
MSM	57 (41.9)	24 (20.2)
Heterosexual	44 (32.4)	70 (58.8)
Drug use*	12 (8.8)	9 (7.6)
Prostitution	1 (0.7)	2 (1.7)
Homosexual/Heterosexual	8 (5.9)	1 (0.8)
Heterosexual/Prostitution	0	12 (10.1)
Heterosexual/Drug use	1 (0.7)	1 (0.8)
Drug use/Piercing	2 (1.5)	0
Heterosexual/Drug use/Piercing	1 (0.7)	0
unk**	10 (7.4)	0
**TOTAL**	**136**	**119**

*It includes both injecting and non-injecting drug users

**At-risk behaviour not defined or unknown
